# Mapping the Landscape of Obesity Effects on Male Reproductive Function: A Bibliometric Study

**DOI:** 10.2174/0118715303271117231220072051

**Published:** 2024-01-30

**Authors:** Yanhong Wei, Meihua Liao, Yiming Lu, Xiaocan Lei, Junli Wang, Xiaoqiong Luo, Linlin Hu

**Affiliations:** 1 Reproductive Medicine, Guangxi Medical and Health Key Discipline Construction Project, The Affiliated Hospital of Youjiang Medical University for Nationalities, Baise, 533000, China;; 2 Clinical Anatomy and Reproductive Medicine Application Institute, Hengyang Medical School, University of South China, Hengyang, 421001, China;; 3 Industrial College of Biomedicine and Health Industry, Youjiang Medical University for Nationalities, Baise, 533000, China

**Keywords:** Obesity, male reproductive disorders, bibliometric analysis, sperm quality, metabolic syndrome, cardiovascular disease

## Abstract

**Background:**

Due to changes in lifestyle and dietary habits, the global population with obesity is increasing gradually, resulting in a significant rise in the number of individuals having obesity. Obesity is caused by an imbalance between energy intake and consumption, leading to excessive fat accumulation, which interferes with normal human metabolism. It is also associated with cardiovascular disease, metabolic syndrome, male reproductive endocrine regulation disorders, systemic and local inflammatory reactions, excessive oxidative stress, and apoptosis. All these factors can damage the internal environment for sperm generation and maturation, resulting in male sexual dysfunction, a decline in sperm quality, and lower fertility. This study analyzes the trends and priorities of the effects of obesity on male reproductive disorders from a bibliometric perspective.

**Methods:**

This study uses the Web of Science as the statistical source, covering all time spans. Tools like Web of Science, VOSviewer, and CiteSpace are used to analyze countries, institutions, authors, journals, and keywords in the field. Total publications, total citations, and average number of citations are selected for statistics.

**Results:**

The results show that the research on the impact of obesity on male reproductive function can be roughly divided into three stages: the initial stage, the slow development stage, and the rapid development stage. Our statistical scope includes 463 highly relevant articles that we have screened. We found that the journal with the most publications in this field is Andrologia, and the institution with the highest total citations is the University of Utah. The most influential countries, institutions, and authors in this field are the United States, the University of Utah, and Carrell, Douglas. Currently, research related to the impact of obesity on male reproduction focuses mainly on three aspects: biochemistry, molecular biology, and reproductive biology. The keyword explosion results indicate that sperm, obesity, and male reproduction are at the forefront and trends of future research in this field. There has been a shift from basic biochemical and molecular research to research on molecular mechanisms relying on omics technologies. However, we have observed that the number of papers published in 2022 is lower than in 2021, indicating a growth interruption during this period. Considering that this deviation may be due to the impact of the COVID-19 pandemic, it may hinder the progress of certain experiments in 2022. In recent years, China has rapidly developed research in this field. However, the average citation rate is relatively low, indicating the need for Chinese scholars to improve the quality of their articles further. Based on our research and in the context of global obesity, men are at risk of increased infertility. Addressing this issue relies on our continued research into the mechanisms of obesity-related male reproductive disorders. Over the past forty-three years, with the contributions of scientists worldwide, research in this field has flourished.

**Conclusion:**

The impact of obesity on male reproductive disorders has been extensively studied. Currently, research in this field primarily focuses on male sperm function, sperm quality, and the effects or mechanisms of cells on male reproduction. Future trends in this field should concentrate on the relationship between male fertility and energy metabolism, as well as the endocrine function of adipose tissue. This study comprehensively analyzes the current research status and global trends in obesity and male reproductive disorders. We also discuss the future developments in this field, making it easier for researchers to understand its developmental history, current status, and trends, providing valuable reference for effective exploration in this area.

## 1 INTRODUCTION

Obesity is a growing public health concern worldwide [1-4]. It is mainly caused by poor lifestyle habits, particularly the excessive consumption of high-energy diets and reduced physical activity [5], resulting in an imbalance between energy intake and consumption. Excessive fat accumulation can negatively impact health, leading to cardiovascular disease [6], type 2 diabetes [7], malignant tumors, neurodegeneration, accelerated aging, and affecting male reproductive endocrine regulation [8]. Obesity can also result in decreased testosterone levels [9], impaired spermatozoa production [10], erectile dysfunction, and a high risk of low sexual desire [11]. Obesity has become a leading cause of low fertility or infertility in men [12-15]. The negative impact of obesity on male reproduction has become a research hotspot in the field of reproductive medicine [16]. This study analyzes the impact of obesity on male reproductive disorders by analyzing the research status and trends of obesity and male reproductive disorders from home and abroad using Web of Science literature. The study reveals the current research hotspots and provides further research and development direction for future obese male reproductive disorders.

Recently, researchers in this rapidly growing field have conducted a series of studies on various aspects of the impact of obesity on male reproductive disorders, including male infertility, semen quality parameters, and serum reproductive hormones [17], the relationship between body mass index and male sperm apoptosis and related factors [18], and the restoration of impaired reproductive capability through dietary changes [19]. However, taking a macroscopic view of the development and changes in the entire research field, especially the evolution of research trends and future developments, remains challenging.

Bibliometrics is an important discipline that uses mathematical and statistical methods for quantitative analysis [20]. It assesses a specific discipline's social and scientific significance during a particular period [21]. The literature data selected for this study come from the web and are widely recognized as high-quality digital literature resources, considered one of the most suitable databases for bibliometric analysis. This helps researchers analyze the research field related to a given scientific issue from a global perspective and guide subsequent work.

Over the past few decades, bibliometric tools have been developed, with Bibliometrix, CiteSpace, and VOSviewer being the most widely used. Their integration has become a common approach in various fields, including the medical field [22-24], biomaterials [25], and food safety research [26], as they can provide more systematic and accurate information. This article attempts to apply bibliometric methods to conduct quantitative analysis based on the current status of publications, author collaborations, research hotspots, and trending topics in the study of the impact of obesity on male reproductive disorders, aiming to provide a comprehensive overview of the field.

## 2 MATERIALS AND METHODS

### 2.1 Data Sources

In this study, the search database was composed of Science Citation Index Expanded (SCI-EXPANDED), Social Sciences Citation Index (SSCI), Conference Proceedings Citation Index-Science (CPCI-S), Web of Science Core Collection Database (WOSCC), Current Chemical Reactions (CCR-EXPANDED), and Index Chemicus (IC) to enhance the search quality.

### 2.2 Retrieval Strategy

Our criteria for including and excluding literature are as follows: Inclusion criteria: (1) Literature publication dates from January 1, 1980, to February 2, 2023. (2) Literature type is limited to articles. (3) Language is restricted to English. Exclusion criteria: (1) Exclude review or opinion articles and conference papers. (2) Exclude duplicated literature. (3) Exclude literature that does not match the subject terms. (4) Exclude literature in languages other than English. To improve the accuracy and recall of the search, this study employed a topic search with the terms TS=(“overweight” OR “adiposity” OR “individual with obesity” OR “obesity”), AND TS=(“Male infertility” OR “Male reproduction” OR “Male Reproductive disorders”) for all years. We conducted our search using subject terms. The search was completed on February 2, 2023.

### 2.3 Research Methods

This study used WOS (Web of Science) as the data source for statistics. We examined the literature and observed that research in this field has been ongoing since 1980. Therefore, our selected time frame covers the entire span from 1980 to 2023, which spans 43 years. Utilizing the analysis and search capabilities of the Web of Science database, we identified relevant literature from 1980 to 2023. We conducted an analysis from various perspectives, including country, institution, author, source publication, keywords, *etc*., to assess the impact of obesity on male reproductive disorders. The obtained data was input into Excel software for descriptive statistical analysis. The obtained literature was added to the result list of the WOS label, and the data was counted. Complete documents of all the obtained and quoted documents were stored in plain text (txt) and analyzed using VOSviewer and CiteSpace to create the relevant knowledge map. The two software packages complemented each other and were used to analyze the development status and trend of obesity and male reproductive disorders.

## 3 RESULTS

### 3.1 Analysis of Global Publication and Citation Trends

**Fig. (1):**
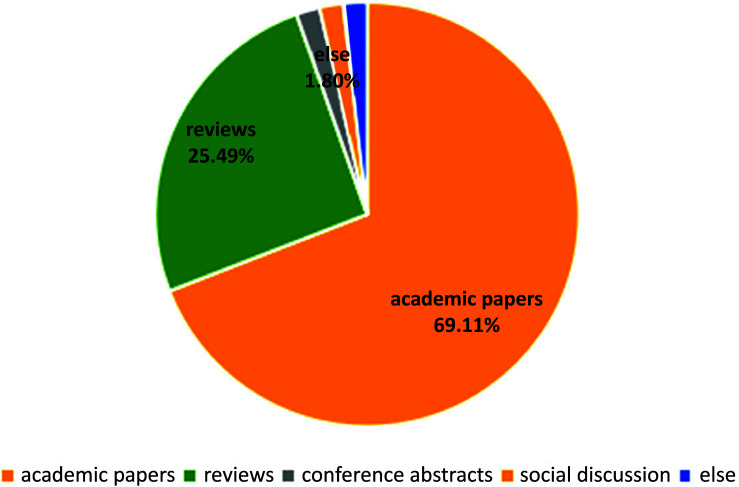
The distribution of literature types in the research field of obesity-related male reproductive disorders. A search conducted within the time span from 1980 to 2023 identified a total of 463 highly relevant publications, with the following classification of literature types: academic papers were the most common (320 papers; 69.11%), followed by reviews (118 papers; 25.49%), and conference abstracts and social gathering materials (25 papers; 5.40%). The literature related to the research topic of this study is primarily composed of academic papers and reviews, accounting for over 90% of the total.

After using the specific keywords mentioned above for the search, we found a total of 5,046 articles. Using CiteSpace to limit the timeframe from 1980 to 2023 and remove duplicate papers, the system displayed 463 highly relevant articles. These articles encompass both clinical experiments and basic experimental research. These papers are contributed by 2,505 authors from 804 institutions in 67 different countries. They were published in 225 journals and have been cited 19,632 times from 3,568 different journals. The majority of these articles are academic papers (320 articles; 69.11%), followed by reviews (118 articles; 25.49%), conference abstracts, and materials from social solicitations (25 articles; 5.40%) (Fig. **1**).

The number of publications related to obesity and male reproductive disorders has been increasing every year, as shown in Fig. (**2**). However, it is worth noting that the number of papers published in 2022 is lower than in 2021, indicating a growth interruption. Considering that there is usually a 1-2 year lag between conducting experiments and publishing papers, this deviation is likely due to the impact of the COVID-19 pandemic, which may have hindered the progress of certain experiments in 2022. Nevertheless, it is clear that over the past forty years, the field of obesity and male reproductive disorders research has evolved significantly.

Based on the annual research output, the growth in the total number of research papers on obesity and male reproductive disorders can be roughly divided into three stages: the initial stage (1980-2006), the slow development stage (2007-2016), and the rapid development stage (2017-2022). Only nine papers were published in the initial stage, primarily focusing on how obesity affects reproductive capabilities. It was not until 2007 that many researchers began to explore other topics related to obesity and reproductive disorders. By 2007, with the establishment of the human genome, transcriptome, and proteome, the Human Metabolite Database (HMDB) was created and officially established. We speculate that the development stages of obesity and male reproductive disorders research are closely related to the establishment of metabolomic databases and the proliferation of omics technologies. Establishing metabolite databases and the continuous advancement of metabolomics technology likely facilitated research on obesity and male reproductive disorders.

However, many scientific questions are difficult to address using a single omics technology. From that point onwards, the development of new omics technologies such as transcriptomics, proteomics, and genomics accelerated the progress in this field.

### 3.2 Analysis of Country/Region Contributions

With economic development and an improved standard of living, obesity and overweight have become the most common health issues worldwide, leading to various health consequences, including some reproductive system disorders. Therefore, research related to the impact of obesity on male reproductive disorders is a globally recognized concern, and scientists from 66 countries have contributed to this field. However, due to various factors, such as different historical and cultural backgrounds related to obesity, changes in agricultural conditions, and economic considerations, contributions from different countries inevitably vary.

**Fig. (3):**
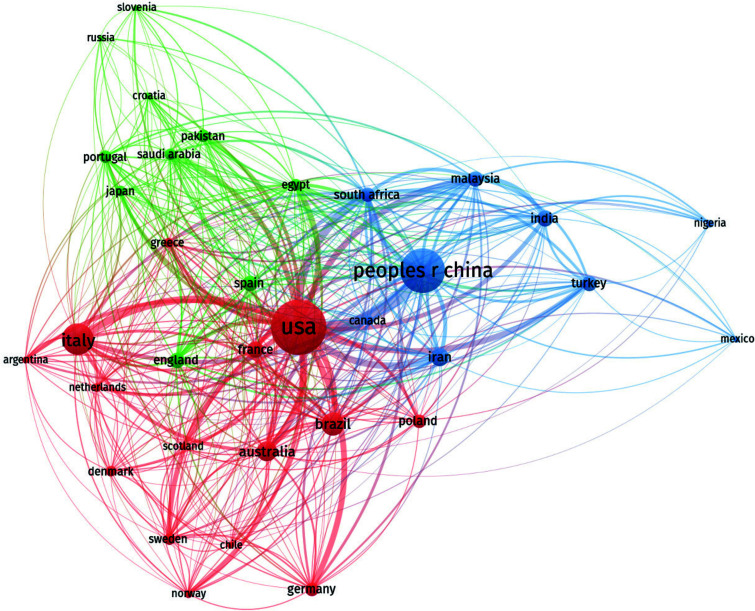
Visual map of the country. In this study, the publication output of 66 countries, with four or more publications, was visualized using VOSviewer. In the figure, larger circular nodes indicate a higher number of publications by a country, the thickness of the connecting lines represents the strength of collaboration between two countries (thicker lines indicate more frequent collaborative publications), and different colors of nodes represent different clusters. From the figure, it is evident that the distribution of countries contributing to publications in this field is highly uneven, with a significant “top-heavy” effect, where the majority of papers are authored by scholars from only a few countries.

In general, countries with a high scientific output in this field are primarily distributed in North America, Asia, and Europe (Fig. **3**), with the United States being a major hub of obesity-related research. In this regard, the United States has published 104 papers over the past forty years, with an average of 66.43 citations per paper, ranking first both in terms of the number of papers and total citations (6,909) (Table **1**). Table **1** lists the top five countries in terms of the number of publications in this field, with China ranking second with 77 articles and 1,076 total citations, but it has the lowest average citation rate among the top five countries. This suggests that Chinese scholars need to continue their efforts in this field.

To determine the countries that have made significant contributions to the study of obesity and male reproductive disorders, we analyzed the number of publications from 66 countries. First, we used VOSviewer to visualize countries with at least four articles (Fig. **3**). The larger the circular node, the higher the number of publications; the connections between nodes represent the strength of their association, with thicker connections indicating more publications between two countries, and the node colors represent different clusters. The United States belongs to the cluster of developed countries where obesity is on the rise due to rapid economic growth and an improved standard of living, leading to increased obesity rates. Therefore, this country places more emphasis on research in the field of obesity, which has resulted in increased financial investments in obesity-related research. This, in turn, has led to universities and research institutions intensifying their research efforts, ultimately contributing to a wealth of research outcomes in this field.

The second most productive country is China, which is a rapidly developing country with increasing levels of development and a growing population, resulting in a rising number of obese individuals and an active presence in this field. However, many other countries, such as Italy, Brazil, and Australia, have also made contributions to research on the impact of obesity on male reproductive disorders, even though they are not major producers in this field. Italy and Brazil have 46 and 29 articles, respectively. The distribution of citations for the top five countries with the highest scientific output encompasses all the papers. While China has contributed some highly-cited papers in this field, the presence of a large number of low-cited literature has brought down the average citation level. This could be related to the rapid growth in research output in China in recent years, as newly published papers often require more time to accumulate citations from other researchers.

### 3.3 Analysis of Institutional Productivity and Co-authorship

**Fig. (4):**
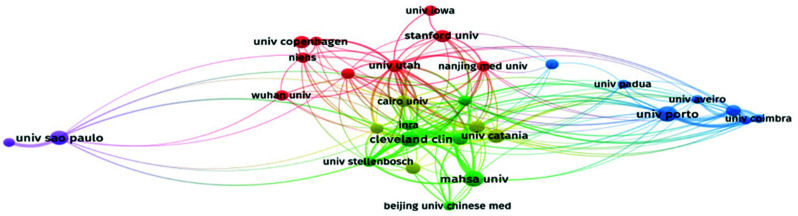
Visualization of the mechanism. Visual analysis was conducted by VOSviewer for institutions with five or more publications, generating a visual map where the size of circular nodes corresponds to the number of publications by each institution. The thickness of the connecting lines between nodes indicates the degree of collaboration between institutions (thicker lines suggest stronger collaboration). Different colors of nodes represent distinct clusters, and the figure displays a total of 30 clusters.

From the perspective of affiliated institutions, universities and public research institutions are the main contributors to research on the impact of obesity on male reproductive disorders, while private enterprises and research institutions have not played a central role. A network of institutional collaboration describes the collaboration between institutions (Fig. **4**), showing that the collaboration between research institutions from different countries is closely interconnected. Although institutions within the same region collaborate more frequently, there is still room for enhanced collaboration. To identify which institutions have made significant contributions in this field, we used VOSviewer to analyze the affiliations of the literature. The results show that 804 institutions have been involved in these reference documents.

Among the top 10 organizations with 8 or more publications, the Cleveland Clinic, MAHSA University, and Porto University have published 11 articles each. However, the University of Utah has received 752 citations, with an average of 94 citations per article, indicating high-quality publications that have garnered significant attention in the field of obesity and male reproductive disorders (Table **2**). An analysis of the papers published by the University of Utah reveals that they primarily publish research papers.

Next, we used VOSviewer to visually analyze institutions with five or more articles (Fig. **4**). The larger the circular node, the more articles are associated with it, and the connections between the two nodes are also larger. Different colors represent different clusters, with a total of 30 clusters in the graph. As shown in Table **2** and Fig. (**4**), universities support the majority of related research institutions, contributing a substantial number of publications.

### 3.4 Analysis of Productivity and Co-authorship of Authors

We further analyzed the top ten authors with the most publications, their citation counts, and related information, as shown in Table **3**. The top three authors in terms of the number of publications and global citation counts are Dutta, Sulagna (with 10 publications and 91 citations), Agarwal, Ashok (with 10 publications and 593 citations), and Sengupta, Pallaw (with 9 publications and 67 citations). However, the most cited author is Douglas Carrell, with 752 citations and an average citation rate of 107.43 times, indicating the high quality of his articles and widespread recognition by authors in this field.

Fig. (**5**) shows a visual map analysis of authors with two or more articles using VOSviewer. According to the annual research output of top authors, some top authors have been active in this field since 2007. Many others joined around 2009 and contributed to the development of research on the impact of obesity on male reproductive disorders. By 2014, nine of the top 10 authors were active in this field, and two years later, research in this area entered a rapid development phase. The analysis of literature authors reveals the representative scholars and core research forces in this research field.

According to Price's law, the minimum number of core authors in a field is given by m = 0.749n_max^2.37 (where n_max = 10), so authors with two or more papers (including two papers) are identified as core authors in this field. There are a total of 277 core authors, accounting for over 50% of the total number of papers, meeting the standard set by Price [27] of at least 50%. When substituting these values into the formula, it aligns with Price's law calculation. Therefore, it can be considered that the research on the impact of obesity on male reproductive disorders has formed a relatively stable author collaboration group.

Table **3** displays prolific authors who have published more than 7 papers in this field. We further examined the collaborative relationships between authors. We found that the trend of elitism is more apparent in terms of authorship than in institutions. Many internally closely connected clusters can be found in the author collaboration network, and the aggregation of these clusters aligns with the authors' affiliations. Authors are divided into multiple clusters within the collaboration network; cooperation is strong within and relatively weak between clusters. For example, authors from the same university, such as Wan Xiaochun, Wei Chaoling, and Deng Weiwei, are very close on the map. Dutta, Sulagna is closely connected with Akhigbe, Roland, Eghoghosoa; Agarwal, Ashok with Henkel Ralf, Eisenberg, Michael L with Del Giudice, and Francesco. Furthermore, Dutta and Sulagna have higher centrality than other authors, indicating that they have a broader influence in the collaboration network of research on the impact of obesity on male reproductive disorders.

### 3.5 Analysis of Highly Influential Journals

**Fig. (6):**
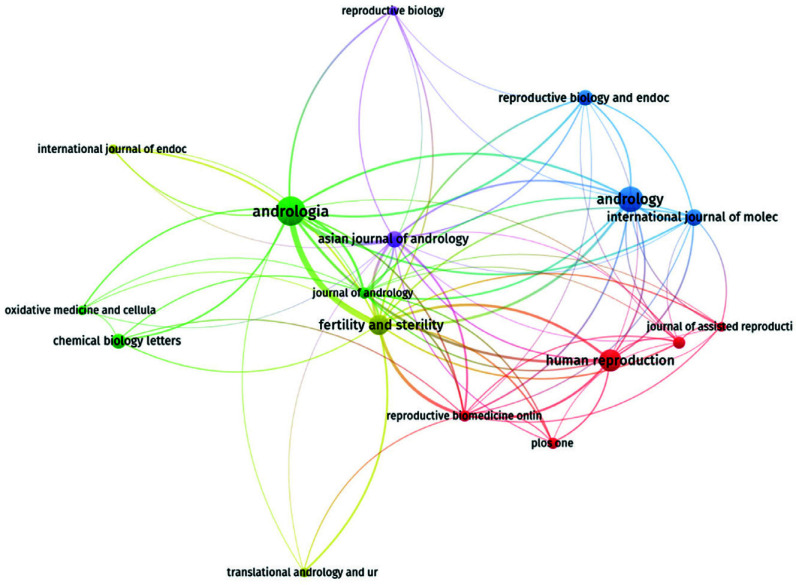
Journal visualization network. Journals with more than 5 articles published were selected to create a visual network map. The clustering is quite apparent, with most of the journals belonging to the medical field, except for a few interdisciplinary journals. Among these journals, there are 25 articles related to androgens, 21 articles related to urology, and 17 articles related to human reproduction. An analysis of journal citations reveals that the “Reproduction and Fertility” journal is highly cited in the medical field, with a total of 15 articles cited 77.33 times, indicating the high quality of articles published in this journal and its significant impact in the field of reproductive medicine.

In addition to a small number of comprehensive journals, most of the journals belong to the medical field. Table **4** shows the top nine journals with the number of articles. Among them are 17 journals with 25, 21, and 17 articles, respectively, in Andrologia, Andrology, and Human Reproduction. The analysis of the citations of journals found that Fertility and Sterility was the most cited journal in the medical field, with a total of 15 articles cited 77.33 times, indicating that the articles published in this journal were of high quality and attracted a lot of attention in the field of reproductive medicine. An analysis of its published literature shows that the journal mainly includes academic papers and focuses on reproductive endocrinology, urology, male science, and other reproductive-related studies. Journals with more than five articles were selected to form a visual network map (Fig. **6**), and the clustering benefits were obvious.

### 3.6 Analysis of Co-occurring Keywords

Analyzing the keyword network is significant in exploring hot spots in a field.

**Fig. (7):**
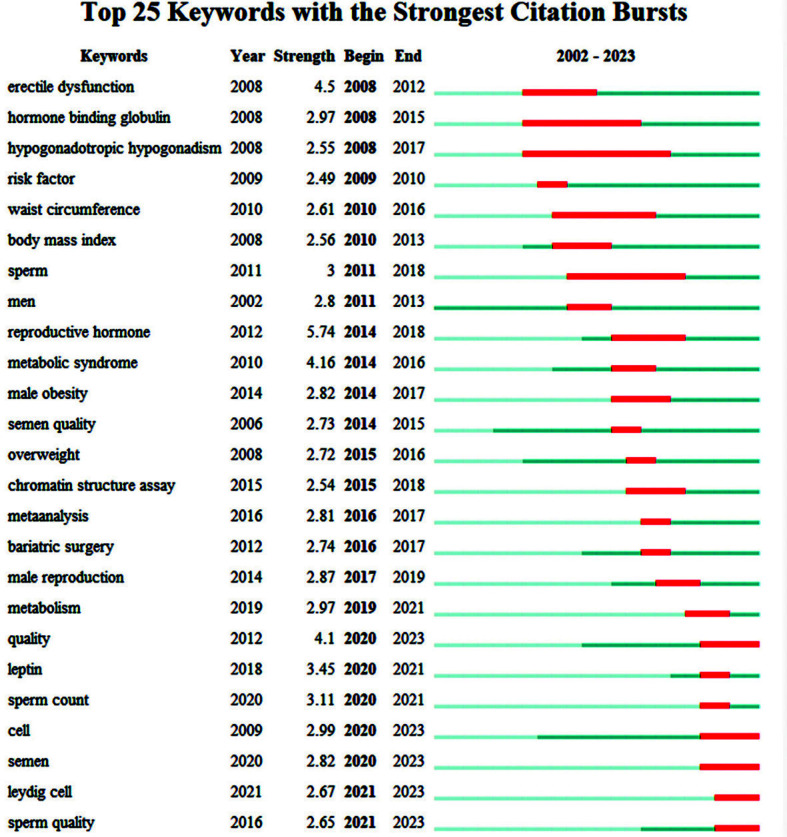
Keyword outbreak diagram. The 25 strongest keywords by the beginning year of their emergence are listed based on the number of citations. This figure displays a network of keywords that co-occur and identifies clusters using VOSviewer. Each node represents a keyword that meets the filtering threshold, and the node's size is related to the number of articles in which that keyword appears. The lines between nodes represent co-occurrence relationships between keywords.

**Fig. (8):**
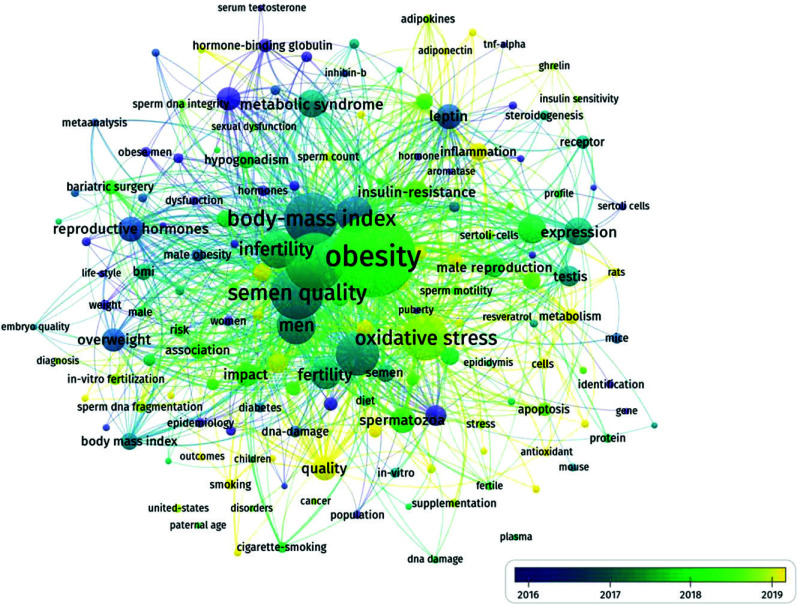
Keyword overlay visualization view. The analysis involved using VOSviewer to examine the source files and identify a total of 2,133 keywords. Keywords that appeared six times or more were selected to create a keyword visualization network, and then CiteSpace was used to generate a keyword burst map. The highest burst strength in the keywords is associated with “obesity” and “male semen quality and infertility.” This is because male reproductive disorders are primarily caused by obesity.

In the past forty years of research on the impact of obesity on male reproductive disorders, we used VOSviewer to generate a keyword co-occurrence network, further revealing clustering relationships and co-occurrence patterns. Using VOSviewer, four clusters were identified, corresponding to different research topics: green, blue, purple, and yellow clusters (Figs. **7** and **8**).

The average citation of generated keywords may suggest that, despite the widespread attention from researchers, there is still room for increasing the quantity of research in these areas. In the more frequently occurring clusters, the most commonly cited keywords are “obesity,” “sperm quality,” and “body mass index.” These keywords are related to the impact of obesity on male reproductive disorders and its regulatory mechanisms, and they are distributed in the green and blue clusters. The green cluster mainly concerns the regulatory mechanisms of obesity and includes keywords with the highest frequency of occurrence. Other high-frequency keywords such as “body mass index,” “obesity,” and “semen quality” are also categorized into the green cluster.

The blue cluster deals with the metabolism and characterization related to males and sperm, where “sperm quality” represents the primary focus of this cluster. Other lower-frequency keywords, including “leptin,” “expression,” “semen,” and “overweight,” encompass the most relevant keywords for the impact of obesity on male reproductive disorders.

The purple cluster's keywords have a generally lower occurrence frequency compared to the other two clusters, but they still convey substantial information related to the impact of obesity on male reproductive disorders. The most common keywords in this cluster include “sperm DNA integrity,” “obese men,” “hormone-binding,” and “weight,” which indicate the primary interests of this cluster.

Keywords such as “reproductive hormone,” “metabolic syndrome,” “erectile dysfunction,” *etc*., exhibit higher frequency and trend status values, signifying their sustained focus in this field. From 2009 to 2015, hot topics included “hormone-binding globulin,” “hypogonadotropic hypogonadism,” “risk factor,” and “waist circumference,” which leaned more towards research on male semen and obesity-related risk factors. Around 2016, interest in this field rapidly expanded to encompass topics such as “sperm quality,” “sperm quantity,” and “Leydig cell.” In the last four years of the past forty years, hot topics like “cell,” “semen,” “Leydig cell,” and “sperm quality” represented a strong focus on the cellular and molecular aspects of the impact of obesity on male reproductive disorders. Moreover, it's worth noting that “cell” and “quality” appeared in 2020 with relatively high frequencies and trend status values, indicating increased attention to these keywords around that time.

The density visualization map of hot topics reflects the development and evolution of research hotspots and interests in the field of obesity's impact on male reproductive disorders. There were too few publications in the initial stage to identify meaningful keywords. In contrast, research topics significantly expanded during the slow development stage. Researchers began by studying obesity phenotypes and topics related to physiology in obese populations, and they quickly developed into molecular biology research related to sperm quality and sperm quantity. As the number of publications increased, the regulatory mechanisms related to sperm production received significant attention during the rapid development stage. Keywords related to sperm quality and quantity became popular topics in the last two to three years, likely due to a series of high-quality articles published recently on the impact of obesity on male reproductive disorders.

## 4 DISCUSSION

This study utilized bibliometric tools to analyze the impact of trends in the field of obesity on male reproductive disorders over the past 40 years, including its development stages, author collaborations, research topics, hotspots, and their temporal evolution. Based on the current publications and research topics, the historical development was divided into three stages: the initial stage, the slow development stage, and the rapid development stage. Among them, the journal with the most published articles was “Andrologia,” and the institution with the highest total citations was the “University of Utah.” The most influential countries, institutions, and authors in this field were the United States, the “University of Utah,” and “Carrell, Douglas.” Cooperation among authors from different institutions was significantly less frequent than cooperation within the same institution. Since scientific collaboration promotes increased scientific output and impact across regions or countries, establishing broad and effective collaborations will be a challenge for advancing this field in the future. Researchers have extensively explored three main themes: the regulation mechanisms of sperm production, the metabolism and characteristics of males with obesity, and the impact of obesity on male reproductive disorders, and they have made significant progress in these areas.

This study aims to analyze the effects of obesity on male reproductive disorders to understand the current research status in this field and provide a reference for future research directions. A total of 463 relevant articles were detected by searching the Web of Science database. The number of literature publications on this topic is increasing, indicating that the global focus on the impact of obesity on male reproductive disorders is growing. The quantity and quality of published literature are important indicators for evaluating the scientific research strength of a country or institution [28]. The United States has published the most articles (104) and has the most institutions (nine) in the top 10, indicating its leading position in the research field of obesity and male reproductive disorders [29]. Nine out of the top 10 institutions with the largest number of documents are universities, which are related to their strong faculty, research atmosphere, and support.

In recent years, the number of published literature on this topic in China ranks second in the world, although there is still a gap with developed countries. This may be related to the late start of research on obesity and reproduction in China, less attention given to the impact of obesity and male reproductive disorders, and the relatively few Chinese literature included in this database. Understanding high-yielding countries and institutions and increasing cooperation with relevant research teams and universities can help Chinese researchers improve their own research level and promote the progress of research on obesity and male reproductive disorders.

A total of 2505 authors participated in the literature on the impact of obesity on male reproductive disorders, with the top three authors being from India, the USA, and Saudi Arabia, indicating that the study of obesity on male reproductive disorders is transregional and receives widespread attention worldwide. According to Price's Law, global research on obesity and male reproductive disorders has formed a core author group, indicating that there is room for further research and that the scope of research can be expanded.

A total of 225 international journals published literature on the effects of obesity and male reproductive disorders, with nine journals containing more than seven articles and the top three journals being from Germany, the United States, and the United Kingdom. In up to 50% of cases, infertility problems originate from males only [30]. According to some data, the quality of human semen has decreased by 50-60% over the last 40 years [31]. High-fat diet and obesity due to an unhealthy lifestyle affect not only sperm structure but also sperm development [32]. In obese individuals, disease in the hypothalamic-pituitary-gonadal axis, increased estrogen levels, and decreased levels of testosterone, luteinizing hormone (LH), and follicle-stimulating hormone (FSH) were observed. Healthy populations are clearly associated with better sperm quality and, a smaller risk of abnormalities, such as sperm count, sperm concentration, and motility, and lower sperm DNA fragmentation [33]. In addition to mineral components such as zinc [34] and selenium [35], the effects of ω -3 fatty acids [36], leptin [37], and other leptins [38], Coenzyme Q10 [39], glutathione [40] and antioxidant vitamin should be emphasized, as their effects are mainly based on the minimizing oxidative stress [41] and inflammatory processes [42]. Improving sleep quality and strengthening exercise can also be therapeutic interventions for patients with obesity and reproductive disorders [43, 44]. It is believed that more international cooperation will occur in the future, and developing countries will play a more important role in making breakthroughs in basic scientific research, bringing good news to patients with obese men with reproductive disorders.

Obesity, infertility, metabolic syndrome, and lifestyle are hot topics in current research. Future research can focus on the status and prognosis of the treatment of obese reproductive disorders in men and strengthen the application of qualitative research and scientific intervention. In this study, we analyzed the characteristics of obesity in male reproductive research by examining the year, country, institutions, authors, publications, and keywords. However, there is a lack of literature sources, research methods, and domestic obesity and male reproductive research literature. Therefore, we hope that future researchers will compare domestic and foreign obesity on male reproductive research differences to guide further research in this field by domestic scholars.

The incidence of male reproductive system diseases caused by obesity is increasing year by year and has become a prominent global issue. Based on our research it will provide a reference value for clinical treatment of obesity-related male reproductive dysfunction and the reduction of obesity incidence through drugs and diet. The male reproductive disorders caused by obesity primarily include effects on cell proliferation and apoptosis [45], oxidative damage [46], effects on the cell cycle [47], inhibition of sperm production [48], effects on testosterone synthesis and secretion [49], and erectile function [50]. We also observed that most studies are conducted in animal models, with limited research involving humans. Therefore, further research using human *in vivo* models is needed. For example, some studies have tested the upregulation of specific miRNAs in obese subjects with male infertility. In addition, research on proteins related to male infertility is essential. For instance, Sertoli cells have been used in recent studies to counteract the harmful effects of metabolic disorders. At the same time, these methods can serve as molecular targets for combating infertility caused by obesity in male patients, entering the preclinical stage and providing a promising treatment opportunity for obesity-related male infertility. According to our analysis, research in the field of obesity-related male reproductive disorders is expected to become an emerging hotspot in the coming years and will contribute to the development of this field. Currently, research on obesity-related male reproductive disorders is mainly limited to cell and animal models [51-53], lacking substantial clinical research data. In the future, comprehensive and in-depth research on obesity-related male reproductive disorders can be conducted through network pharmacology, bioinformatics, multi-omics analysis, *etc*., while gradually conducting clinical trials further to elucidate the impact of obesity on the body, promoting obesity research to play a more important role in male reproductive health.

## CONCLUSION

The impact of obesity on male reproductive disorders has been extensively studied. Currently, research in this field primarily focuses on male sperm function, sperm quality, and the effects or mechanisms of cells on male reproduction. Future trends in this field should concentrate on the relationship between male fertility and energy metabolism, as well as the endocrine function of adipose tissue. This study comprehensively analyzes the current research status and global trends in obesity and male reproductive disorders. We also discuss the future developments in this field, making it easier for researchers to understand its developmental history, current status, and trends, providing valuable reference for effective exploration in this area.

## Figures and Tables

**Fig. (2):**
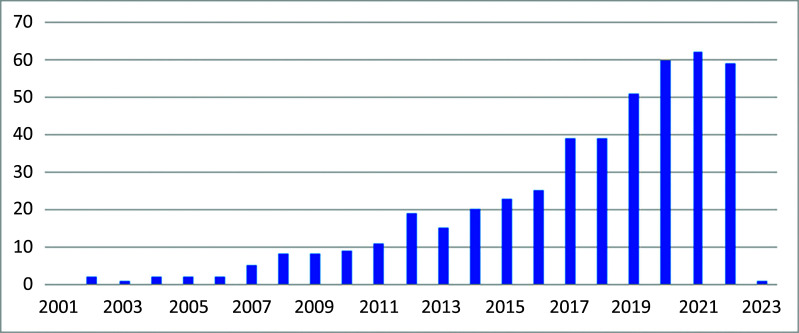
The research progress in the field of obesity-related male reproductive disorders. The number of publications in these studies has shown a general increasing trend over the years. The dataset used in this study did not have any published articles before 2002, and there was a decrease in the number of papers published in 2022 compared to 2021, which interrupted the growth trend.

**Fig. (5):**
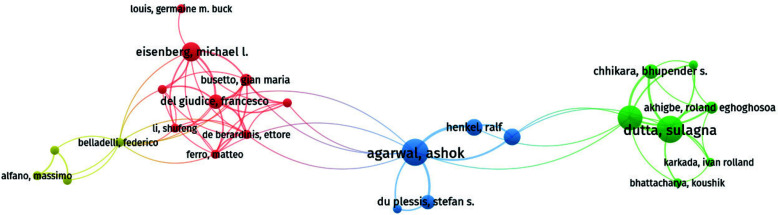
Collaborative network of 277 core authors. Visual analysis was conducted by VOSviewer for authors with two or more publications, generating a visual map. The top three authors with the highest number of publications and total citations (global total citations) are Dutta, Sulagna (with 10 publications and 91 citations), Agarwal, Ashok (with 10 publications and 593 citations), and Sengupta, Pallaw (with 9 publications and 67 citations). However, the most cited author is Douglas Carroll, with 752 citations and an average citation rate of 107.43 times, indicating the high quality of his articles and wide recognition in the field.

**Table 1:** The top five countries in terms of publications.

**Rank**	**Country**	**Publications**	**Citations**	**Average Citation/Publication**
1	The United States	104	6909	66.43
2	China	77	1076	13.97
3	Italy	46	1004	21.83
4	Brazil	29	425	14.66
5	Australia	24	1118	46.58

**Note:** Table shows the countries that have the highest number of publications in this field. According to the data in the analysis table, scholars from the United States have contributed the most research papers in this field, with a total of 104 papers published and an average citation count as high as 66.43 times. Next is China, with a total of 77 publications and 1076 citations.

**Table 2:** Ranking table of institutions with more than 8 articles.

**Rank**	**Organization**	**Documents**	**Citations**	**Average Citation/Publication**
1	Cleveland Clinic	11	675	61.36
2	MAHSA University	11	128	11.64
3	University of Porto	11	210	19.09
4	University of Sao Paulo	10	92	9.2
5	University of Catania	9	192	21.33
6	University of the western cape	8	393	49.13
7	University of Utah	8	752	94
8	Shanghai Jiao Tong University	8	151	18.88
9	Stanford University	8	252	31.5
10	University Copenhagen	8	561	70.13

**Note:** Table lists the top ten institutions with the highest number of publications. Among them, the institution with the most publications is Cleveland Clinic, MAHSA University, and the University of Porto, each having 11 publications. However, the University of Utah has the highest total citation count, reaching 752, with an average of 94 citations per paper.

**Table 3:** High productivity authors with more than 7 posts.

**Rank**	**Author**	**Documents**	**Citations**	**Average Citation/Publication**
1	Dutta, Sulagna	10	91	9.1
2	Agarwal, Ashok	10	593	59.3
3	Sengupta, Pallaw	9	67	7.44
4	Alves, Marcog	9	143	15.89
5	Oliveira, Pedrof	9	143	15.89
6	Araujo Leite, Gabriel adan	7	66	9.43
7	Kempinas, Wilma De Grava	7	66	9.43
8	Carrell, Douglast	7	752	107.43

**Note:** Table shows the top ten authors with the highest number of publications. Among these authors, the ones with the most publications are from Cleveland Clinic, MAHSA University, and the University of Porto, each having 11 publications. However, the most cited author is Douglas Carrell, with a total of 752 citations and an average of 94 citations per paper.

**Table 4:** Table of the top 9 journals in terms of publications.

**Rank**	**Source**	**Publications**	**Citations**	**Average Citation/Publication**
1	Andrologia	25	584	23.36
2	Andrology	21	543	25.86
3	Human Reproduction	17	1036	60.94
4	Fertility and Sterility	15	1160	77.33
5	Asian Journal of Andrology	11	398	36.18
6	International Journal of Molecular Sciences	11	126	11.45
7	Reproductive Biology and Endocrinology	10	226	22.6
8	Chemical Biology Letters	9	52	5.78
9	Frontiers in Endocrinology	7	22	3.14

**Note:** Table displays journals with publication counts greater than or equal to 17 papers. Among these journals, Andrologia, Andrology, and Human Reproduction have 25, 21, and 17 publications, respectively.

## Data Availability

The data and supportive information are available within the article.

## LIST OF ABBREVIATIONS

CCR-EXPANDED: Current Chemical Reactions
CPCI-S: Conference Proceedings Citation Index-Science
HMDB: Human Metabolite Database
IC: Index Chemicus
SCI-EXPANDED: Science Citation Index Expanded
SSCI: Social Sciences Citation Index
WOSCC: Web of Science Core Collection Database
